# Immune Inflammation at the Crossroads of Atherosclerosis and Ischemic Stroke: Mechanisms, Trends, and Translational Perspectives

**DOI:** 10.1002/cns.70712

**Published:** 2025-12-28

**Authors:** Hongdong Hao, Dian Chen, Cheng Qian, Xuanyi Zhou, Xi Peng, Guanlin Wang, Jingjing Tang, Hai‐Xin Liu

**Affiliations:** ^1^ Shanxi University of Chinese Medicine Taiyuan China

**Keywords:** atherosclerosis, bibliometric analysis, immune‐mediated inflammation, ischemic stroke, translational immunology

## Abstract

**Background:**

Atherosclerosis is a chronic inflammatory disorder and a major cause of ischemic stroke. Immune‐mediated mechanisms are increasingly recognized as central in this continuum, yet the global research landscape and its clinical translation remain insufficiently characterized.

**Methods:**

We conducted a multi‐level bibliometric analysis using the Web of Science Core Collection and MEDLINE. Searches targeted atherosclerosis, ischemic stroke, and immunity, restricted to English‐language articles and reviews. After screening, 1760 WoSCC records and 708 human‐only MEDLINE articles were analyzed with VOSviewer, CiteSpace, and Bibliometrix. Comparative assessment between China and the United States examined differences in research output, thematic focus, and methodological orientation.

**Results:**

Global publications rose steadily from 1999 to 2025, peaking in 2022. Inflammation, atherosclerosis, and ischemic stroke were the dominant themes, with growing interest in causal inference (e.g., Mendelian randomization) and translational biomarkers. China showed rapid post‐2015 growth with focus on immune‐cell mechanisms, while the United States maintained leadership in scholarly impact, clinical orientation, and collaboration. Human‐only studies confirmed these patterns and highlighted emerging topics such as microRNAs, COVID‐19, insulin resistance, and lipoprotein(a).

**Conclusions:**

Research has shifted from associative links to mechanistic insights and early translational strategies. However, gaps remain between molecular and clinical domains, and causal pathways are underdeveloped. Future work should emphasize molecular–clinical integration, expand immunological targets, apply multi‐omics and AI approaches, and strengthen international collaboration—particularly between China and the United States—to advance precision prevention and intervention in atherosclerotic ischemic stroke.

## Graphical abstract

As requested, we provide the graphical abstract text below. Once approved, we will insert it into the manuscript (Word file):“This review outlines the immune–inflammatory interactions linking atherosclerosis and ischemic stroke, highlights emerging mechanistic pathways, and summarizes potential translational targets across both vascular diseases.”

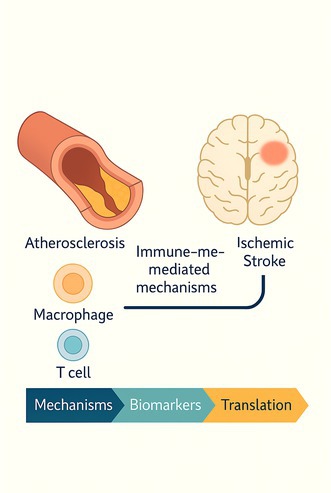

**Abbreviation**
WOSCC: Web of Science

## 1 Introduction

Atherosclerosis is a systemic inflammatory disease and a principal cause of ischemic stroke, the leading cause of long‐term disability [1] and the second most common cause of death worldwide [2]. Over the past two decades, a growing body of research has emphasized the pivotal role of immune‐mediated mechanisms [3, 4]—including innate and adaptive immunity, cytokine signaling, and inflammatory cell activation—in driving the pathophysiological continuum from plaque formation to cerebral infarction [5, 6]. Despite substantial progress, our understanding of how immune responses orchestrate this vascular–neurological axis remains fragmented, often lacking an integrative framework that connects molecular mechanisms with clinical outcomes.

To bridge this conceptual gap, we incorporated human‐only studies from MEDLINE to emphasize clinical relevance and ensure translational alignment with patient‐oriented research.

Moreover, translational insights from experimental immunology have not been fully bridged to population‐level stroke prevention strategies. While inflammation‐related biomarkers (e.g., C‐reactive protein, interleukin‐6) have been investigated in both cardiovascular and cerebrovascular contexts, causal relationships remain incompletely defined, and clinically actionable targets are scarce. Recent methodological advances such as Mendelian randomization [7] and high‐throughput omics offer new opportunities to unravel these complex links [8, 9, 10]. Yet, the field lacks a comprehensive synthesis that maps the evolving research landscape, cross‐national contributions, and the extent of clinical translation.

To address this gap, we conducted a multi‐layered bibliometric analysis to evaluate global trends and thematic trajectories in immune‐mediated research connecting atherosclerosis and ischemic stroke. This study systematically triangulates three analytical levels: (1) global scientific output based on the Web of Science Core Collection; (2) a comparative analysis of China and the United States as leading contributors; and (3) a clinical relevance assessment using human‐only studies from MEDLINE. By integrating co‐authorship networks, thematic clustering, conceptual mapping, and temporal trend analyses across platforms, we aim to delineate research evolution, identify underexplored domains, and propose strategic directions for future translational research.

## 2 Methods

### 2.1 Data Sources and Search Strategy

A comprehensive bibliometric analysis was conducted using the Web of Science Core Collection (WoSCC) and MEDLINE databases. The literature search was designed to capture global research related to immune‐mediated mechanisms connecting atherosclerosis and ischemic stroke. The search strategy combined three major thematic clusters using Boolean operators: (“Atherosclerosis” OR “Arteriosclerosis” OR “Plaque” OR “Coronary Artery Disease”) AND (“Ischemic Stroke” OR “Cerebral Infarction” OR “Brain Infarction”) AND (“Immune” OR “Immunity” OR “Inflammation” OR “Immune Response” OR “Immune Dysregulation” OR “Cytokines” OR “Innate Immunity” OR “Adaptive Immunity”).

The literature search covered publications from January 1, 2020, to August 31, 2025. MEDLINE records were retrieved via the PubMed platform. Filters were applied to include only English‐language articles and reviews. After de‐duplication and screening, 1760 records from WoSCC and 708 human‐only records from MEDLINE were retained for downstream analysis.

Although a precise overlap between the WoSCC (*n* = 1760) and MEDLINE (*n* = 708) datasets was not quantitatively assessed, manual screening suggested partial redundancy mainly among review‐type publications. Therefore, the MEDLINE subset was treated as a clinically focused complement rather than duplication of the WoSCC dataset.

Although the inclusion period began in 2020, earlier years (e.g., 1999–2019) may appear in figures derived from co‐citation and burst analyses because these tools display the historical publication years of influential cited references rather than the publication years of the retrieved dataset.

### 2.2 Screening and Data Extraction

All retrieved records were independently screened by two reviewers (Reviewer A and Reviewer B). Inclusion was based on topic relevance determined by title and abstract review. Discrepancies or ambiguities were resolved through consensus, with arbitration by a third senior reviewer when necessary. Final datasets were exported in plain text and BibTeX formats for subsequent analysis.

### 2.3 Analytical Tools and Indicators

Four analytical platforms were used: VOSviewer, CiteSpace, Bibliometrix (with the Biblioshiny interface), and Microsoft Excel.

For VOSviewer [11], the minimum keyword co‐occurrence threshold was adjusted between 5 and its integer multiples to ensure that each clustering map contained approximately 60–90 representative keywords. Normalization was performed using the association strength method with a default clustering resolution = 1.0.

For CiteSpace, parameters were defined as: time slice = 1 year, term source = title/abstract/keywords, selection criteria = top 50 per slice, using log‐likelihood ratio (LLR) clustering. The k value was flexibly set to 25 or 50 depending on visualization clarity, as larger k values produced denser but less distinguishable clusters. The threshold of top 50 terms per slice was chosen to balance granularity and interpretability, after testing alternative cutoffs (top 30 and top 100) that yielded comparable thematic structures.

Bibliometrix [12] and the Biblioshiny interface were used for comprehensive performance analysis, thematic mapping, and conceptual structure modeling via Multiple Correspondence Analysis (MCA).

Microsoft Excel was applied for data cleaning, manual coding, frequency tabulation, and descriptive statistical analysis.

Earlier publication years shown in keyword and reference burst visualizations represent the temporal distribution of cited literature identified by CiteSpace, not the actual retrieval window (2020–2025) of the analyzed records.

### 2.4 Outcome Metrics

**FIGURE 1:**
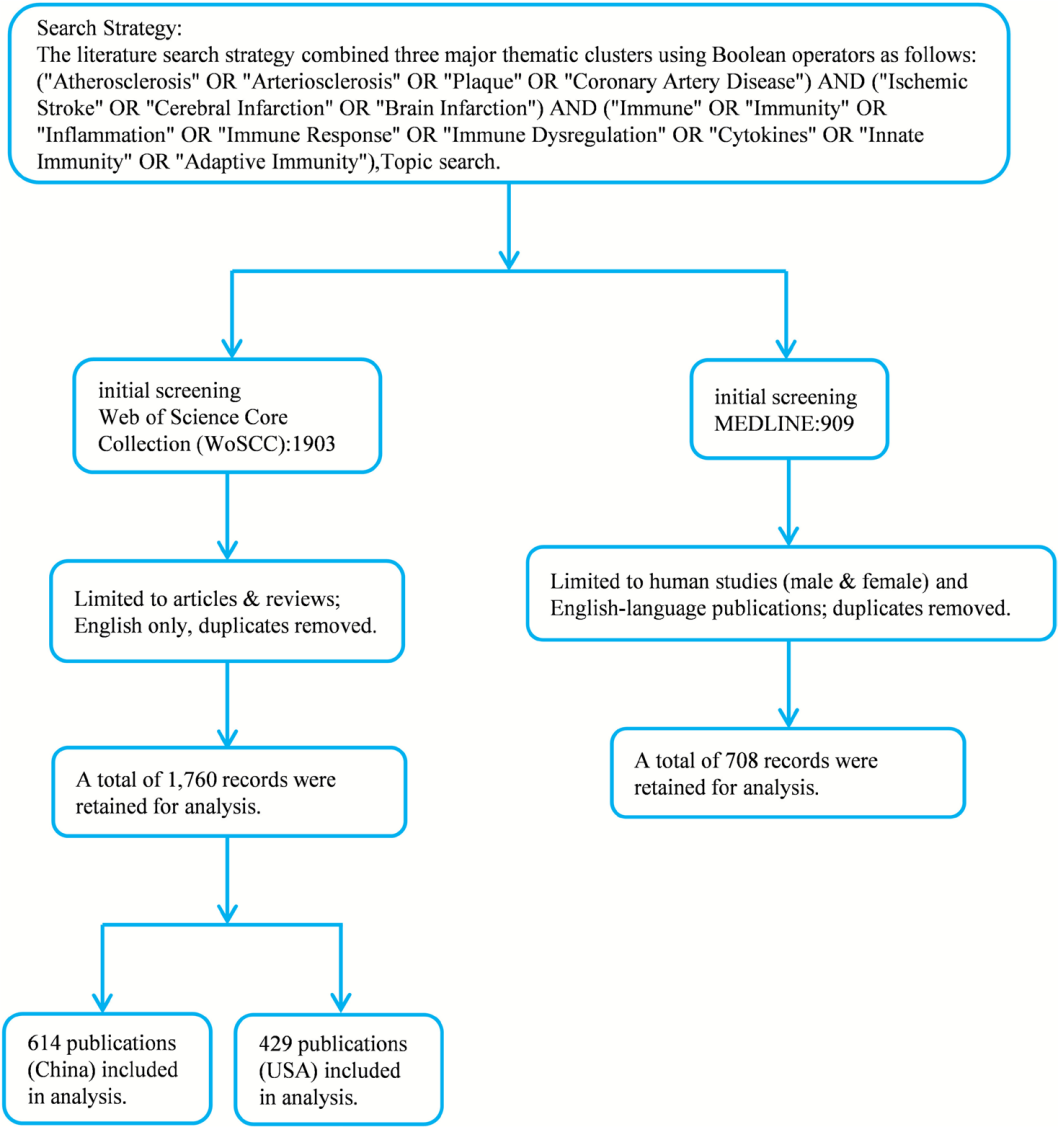
Literature Screening Flowchart.

The primary bibliometric indicators included:

- Publication trends over time
- Authorship and institutional collaboration networks
- Keyword co‐occurrence frequency and clustering
- Citation analysis
- Thematic evolution and conceptual structures
- Country‐specific comparisons (China vs. United States)
- Clinical relevance assessment via MEDLINE human‐only subset (Figure 1).

## 3 Results

### 3.1 Global‐Level Research Landscape and Trends

#### 3.1.1 Bibliometric Characteristics and Global Publication Trends

Bibliometric statistics indicate that a total of 1760 publications were included in this study, sourced from 603 journals and authored by 9763 individuals, among whom 36 published single‐authored papers. The level of collaboration was high, with an average of 7.67 co‐authors per article and an international co‐authorship rate of 21.99%. A total of 2981 author keywords and 72,110 references were identified. The average number of citations per document was 39.4, and the mean publication age was 8.35 years, suggesting sustained scholarly relevance and citation activity in the field.

**FIGURE 2:**
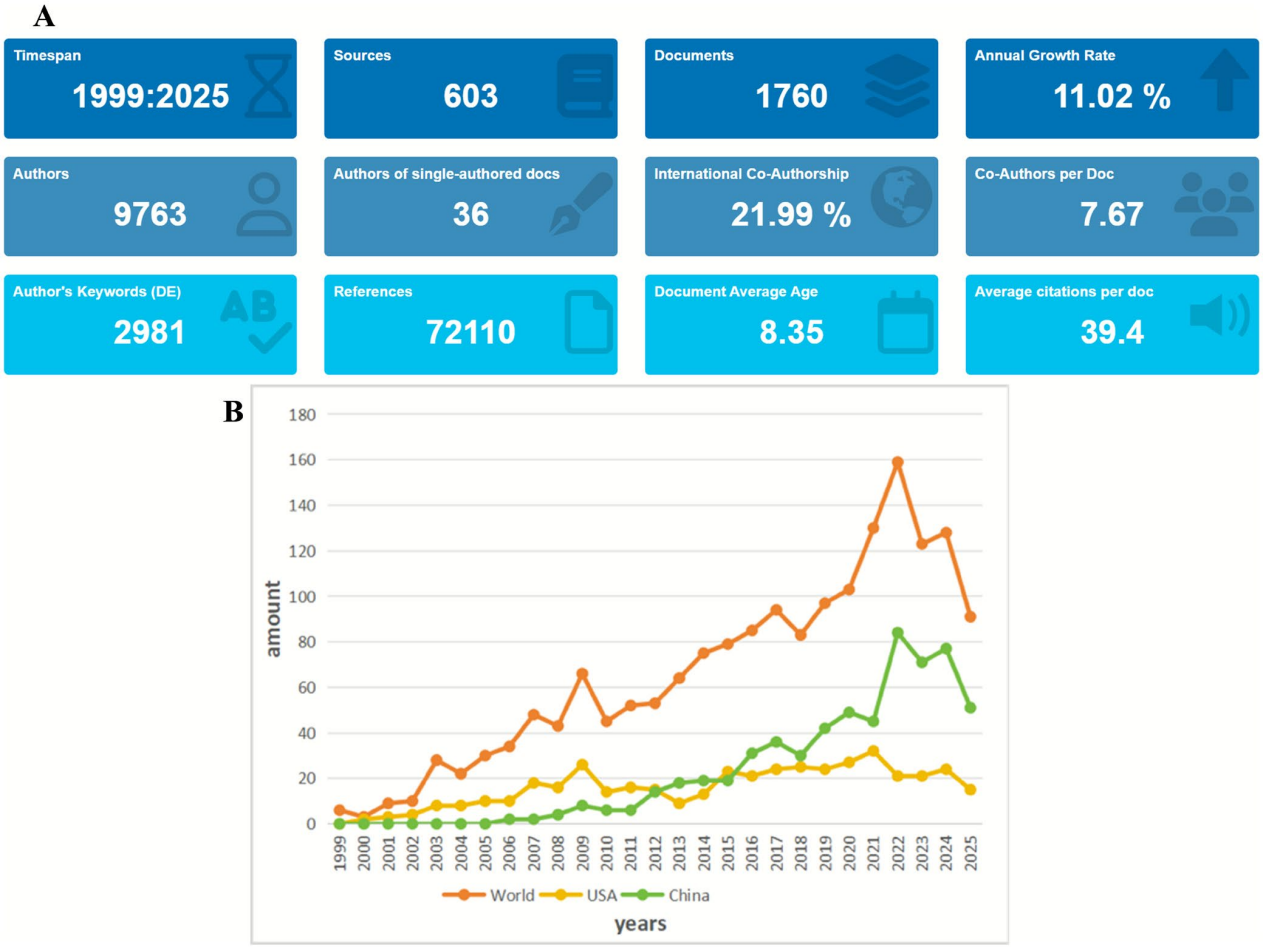
Bibliometric summary and annual publication trends.

Publication trends from 1999 to 2025 reveal a consistent global growth, with an average annual increase of 11.02%. The number of publications peaked in 2022 at 160, followed by a slight decline. By country, China has exhibited a marked increase in output since 2015 and surpassed the United States in 2022 to become the second‐largest contributor in terms of annual publications. The United States maintained steady growth between 2005 and 2015, though its recent publication rate has stabilized (Figure 2).

#### 3.1.2 Geographic, Institutional, and Author‐Level Contributions

At the country level, China (614 articles, 10,083 citations, 107 total link strength) and the United States (429 articles, 28,942 citations, 323 total link strength) were the top two contributors, together forming the core of the global academic collaboration network. Germany (111 articles, 6647 citations, 158 total link strength), the United Kingdom (109 articles, 7782 citations, 186 total link strength), and Italy (117 articles, 6080 citations, 109 total link strength) followed as key European contributors.

At the institutional level, Harvard University (24 articles, 4684 citations, 23 total link strength) ranked highest, followed by Johns Hopkins University (18 articles, 1534 citations, 35 total link strength) and Huazhong University of Science and Technology (27 articles, 680 citations, 13 total link strength).

**FIGURE 3:**
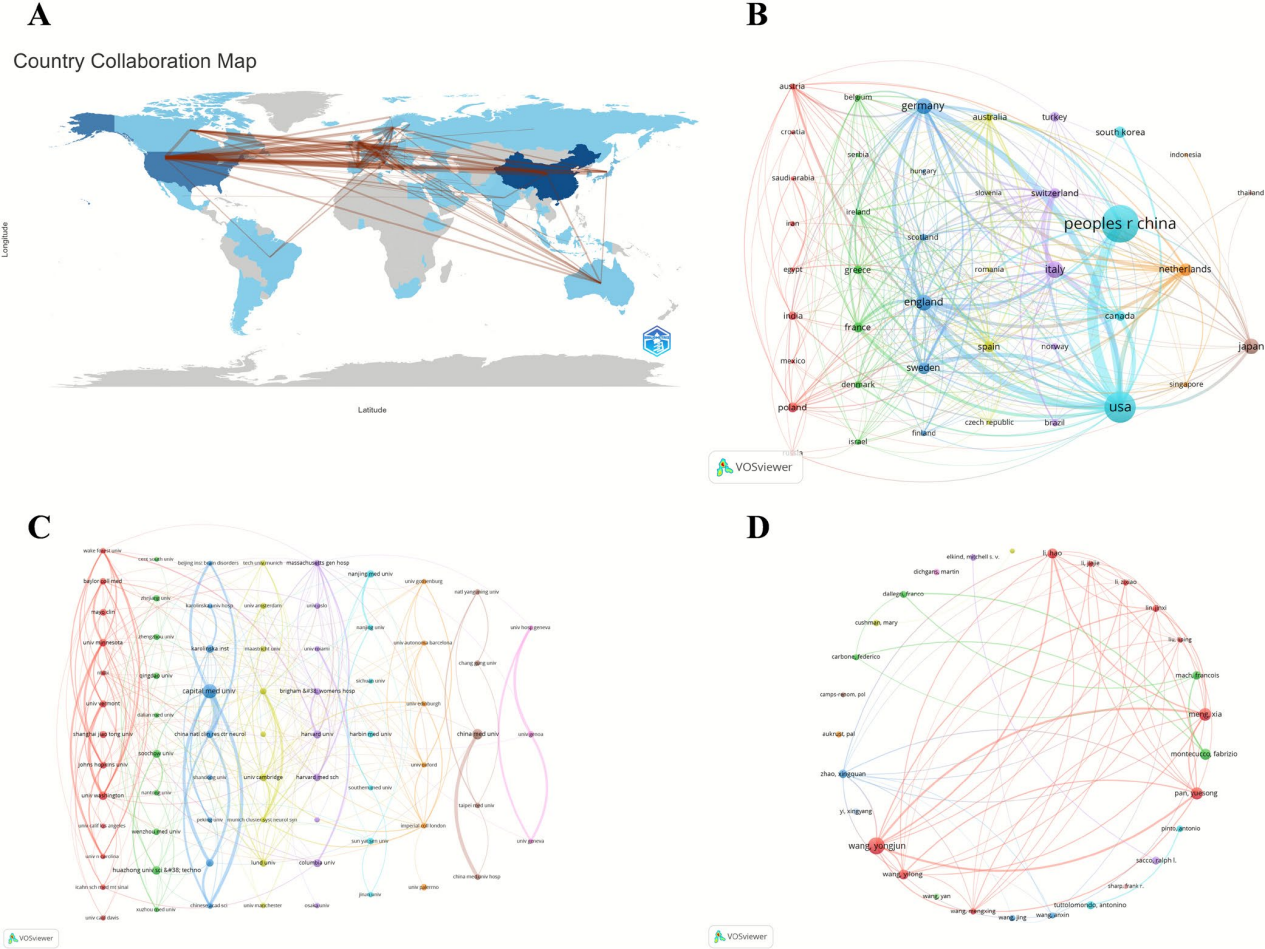
International, institutional, and author collaboration networks. (A) International collaboration map. (B) International collaboration network. (C) Institutional collaboration network. (D) Author collaboration network.

At the individual level, Wang Yongjun (25 articles, 298 citations, 121 total link strength) led, followed by Wang Yilong (14 articles, 238 citations, 73 total link strength) and Meng Xia (18 articles, 234 citations, 95 total link strength) (Figure 3).

### 3.2 Keyword Co‐Occurrence Patterns and Thematic Evolution

**FIGURE 4:**
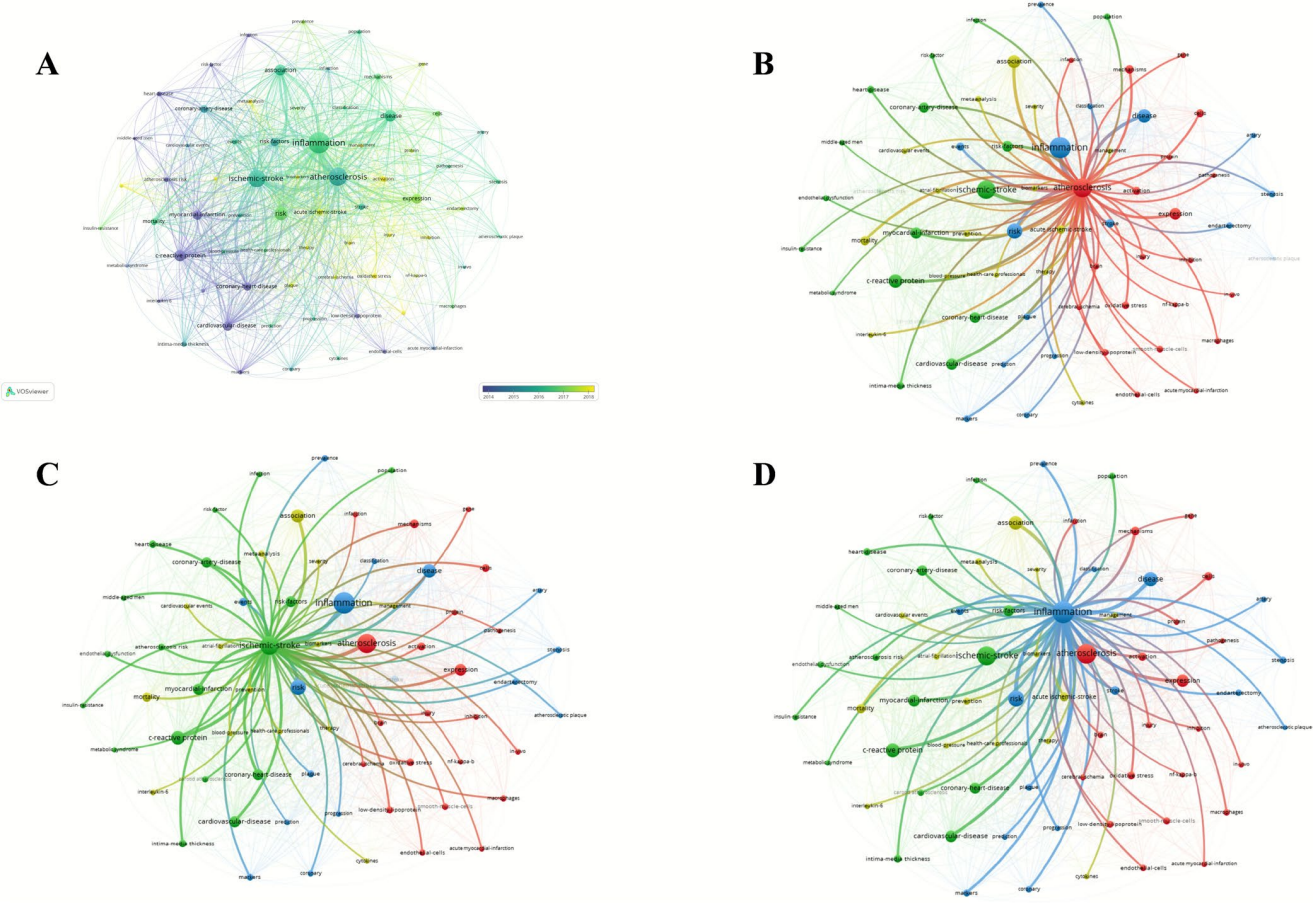
Keyword co‐occurrence and relevance networks. (A) Keyword co‐occurrence network. (B) Relevance network centered on “atherosclerosis.” (C) Relevance network centered on “ischemic stroke.” (D) Relevance network centered on “inflammation.”

Keyword analysis identified *inflammation* (702 occurrences, total link strength: 2539) as the central high‐frequency term, forming a dense co‐occurrence network with *atherosclerosis* (554, 2043), *ischemic stroke* (508, 1838), *c‐reactive protein* (262, 1104), *myocardial infarction* (185, 678), and *cardiovascular disease* (184, 766). Citation burst analysis revealed a thematic shift from early focuses on *coronary heart disease*, *chlamydia pneumoniae*, and *helicobacter pylori* to emerging topics including *Mendelian randomization* (2021–2025, 8.76) and *acute ischemic stroke* (2021–2025, 7.76). Cluster analysis delineated three major thematic structures: a red cluster centered on *atherosclerosis* and mechanistic terms (e.g., *oxidative stress*, *low‐density lipoprotein*); a green cluster focused on *ischemic stroke* and comorbidities (*cardiovascular disease*, *myocardial infarction*); and a blue cluster organized around *inflammation*, linked to epidemiological terms (*risk factors*, *association*, *management*). Temporal overlay mapping showed high‐frequency emergence of *inflammation*, *atherosclerosis*, and *ischemic stroke* during 2014–2018, followed by increased emphasis on Mendelian randomization in more recent years (Figure 4).

Co‐occurrence clustering identified eight principal themes: #0 (endothelial cells) on vascular injury mechanisms; #1 (cells) on inflammatory activation; #2 (C‐reactive protein) on inflammation‐related outcomes; #3 (Mendelian randomization) on causal inference; #4 (cardiovascular disease) on epidemiological patterns; #5 (myocardial infarction) on metabolic–vascular links; #6 (cerebral infarction) on population and subtype research; and #7 (Parkinson's disease) on genetic studies in neurodegeneration.

Cluster #7 (Parkinson's disease) appeared as an isolated thematic outlier, likely reflecting cross‐disease studies exploring shared neuroinflammatory pathways rather than a central research focus. Its inclusion highlights the broader overlap between cerebrovascular and neurodegenerative inflammation.

Citation burst detection identified 25 keywords with strong citation intensity (1999–2025), transitioning from early emphasis on pathophysiological and infectious terms (e.g., *coronary heart disease*, *c‐reactive protein*) to mid‐stage clinical entities (*myocardial infarction*, *carotid atherosclerosis*), and recent focus on *management*, *severity*, *cardiovascular risk factors*, *oxidative stress*, *Mendelian randomization*, and *epidemiology*.

**FIGURE 5:**
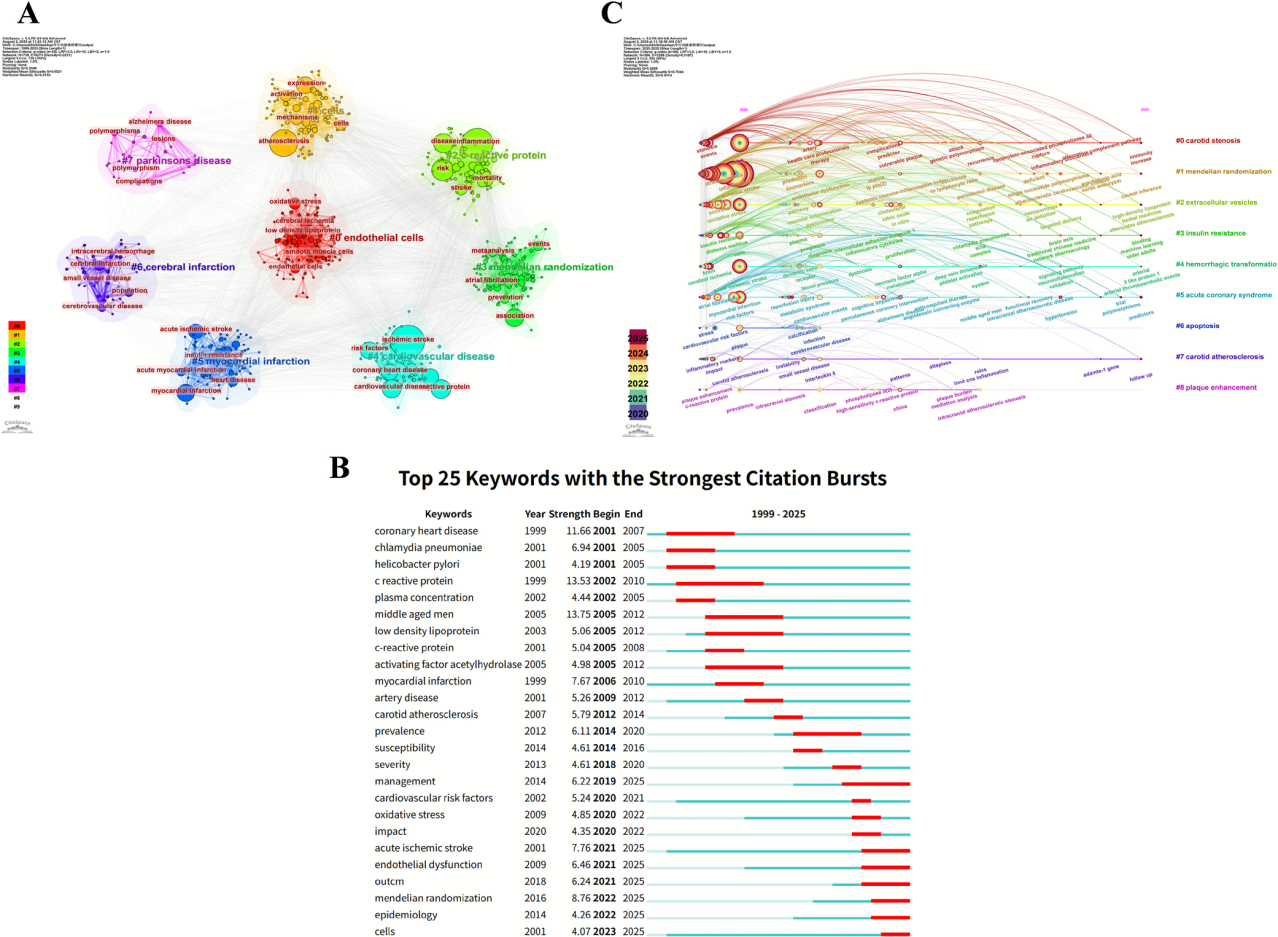
Keyword co‐occurrence, citation bursts, and temporal evolution. (A) Keyword co‐occurrence clusters. (B) Top 25 keywords with the strongest citation bursts (1999–2025). (C) Temporal evolution of keyword clusters.

Between 2020 and 2025, timeline clustering highlighted nine active domains: #0 (*carotid stenosis*) with sustained relevance; #1 (*Mendelian randomization*) emerging from 2022; #2 (*extracellular vesicles*) increasing post‐2021; #3 (*insulin resistance*) growing from 2020; #4 (*hemorrhagic transformation*) active during 2020–2023; #5 (*acute coronary syndrome*) with continuous research presence; #6 (*apoptosis*) peaking around 2020; #7 (*carotid atherosclerosis*) during 2020–2023; and #8 (*plaque enhancement*) during 2020–2022. Overall, thematic evolution has progressed toward mechanistic elucidation, biomarker discovery, and genetic causal inference (Figure 5).

### 3.3 Conceptual Structure and Thematic Maturity of Research Topics

The conceptual structure map, derived from Multiple Correspondence Analysis (MCA), delineates the functional distribution of keywords across two principal dimensions. The lower‐left quadrant encompasses metabolism and vascular‐related terms (e.g., “low‐density lipoprotein”, “coronary‐artery‐disease”); the lower‐right highlights molecular mechanisms (e.g., “oxidative stress”, “macrophages”); the central region aggregates core terms (e.g., “inflammation”, “ischemic stroke”); the upper‐right reflects epidemiological topics (e.g., “mortality”, “meta‐analysis”); and the upper section presents traditional risk indicators (e.g., “atherosclerosis risk”).

The keyword treemap identifies “inflammation” (702 occurrences, 12%) as the most frequent term, followed by “atherosclerosis,” “ischemic stroke,” “risk,” and “c‐reactive protein,” forming the central high‐frequency cluster. Additional keywords related to cardiovascular and cerebrovascular pathology reveal a multidimensional framework anchored in inflammatory processes.

The thematic map classifies research themes by centrality and density: motor themes (e.g., “atherosclerosis,” “inflammation”) exhibit high relevance and maturity; basic themes (e.g., “cardiovascular disease,” “risk factors”) show broad connectivity with moderate development; niche themes (e.g., “genetic polymorphism”) demonstrate high density with limited centrality; and emerging or declining themes (e.g., “polymorphism”) occupy peripheral positions. Transitional topics such as “neuroinflammation” and “COVID‐19” are located near the central intersection.

**FIGURE 6:**
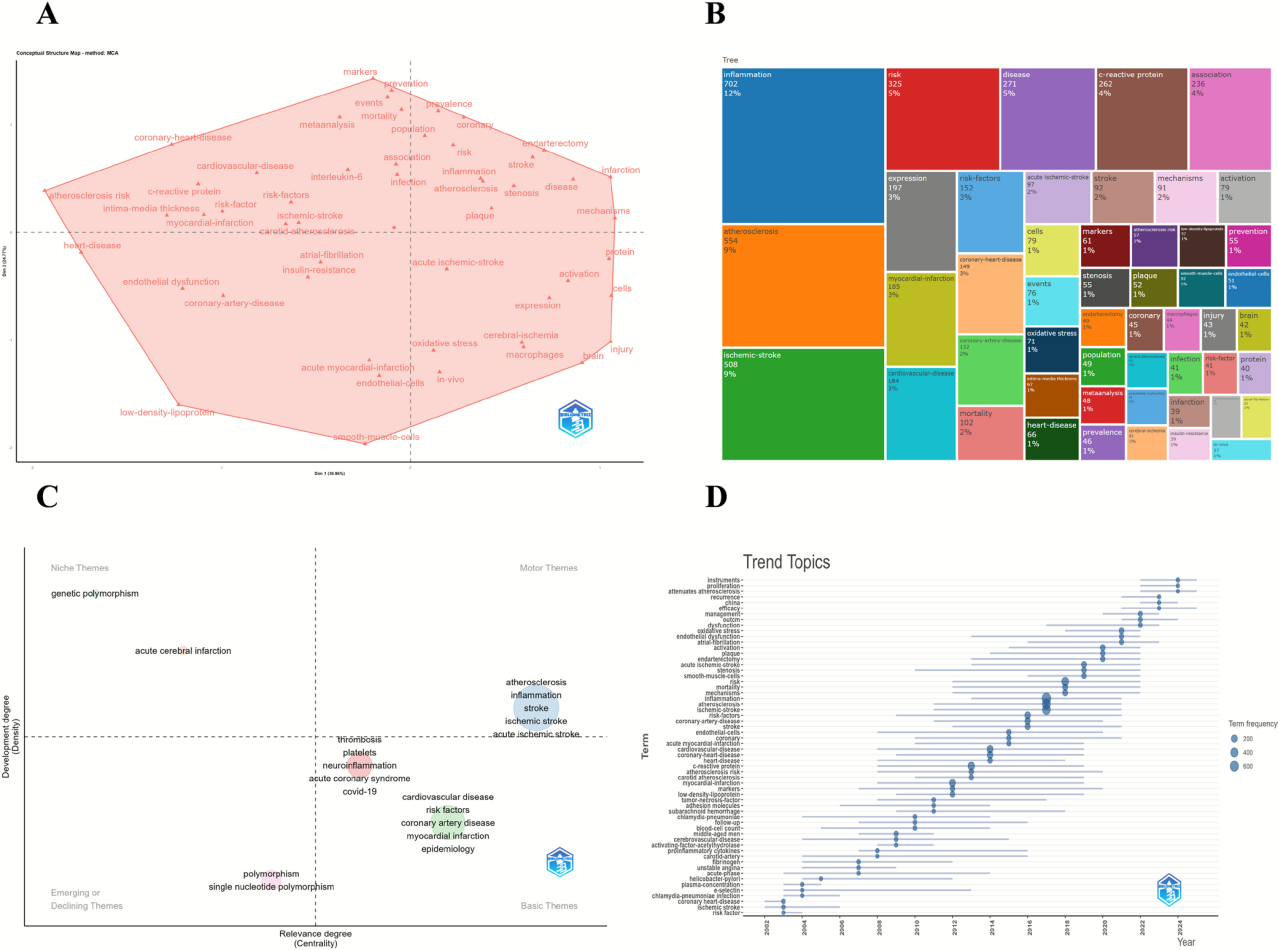
Conceptual structure, thematic distribution, and topic trends. (A) Conceptual structure map based on Multiple Correspondence Analysis (MCA). (B) Keyword tree map. (C) Thematic map. (D) Trend topics over time.

The trend topic map traces the temporal evolution of key terms from 2000 to 2025. Between 2000 and 2010, emphasis was placed on infection and lipid metabolism (e.g., “coronary heart disease,” “helicobacter pylori”); around 2010, focus shifted to mechanisms and disease phenotypes (e.g., “oxidative stress,” “ischemic stroke”); post‐2015, attention increased toward cerebrovascular mechanisms (e.g., “activation,” “atrial fibrillation”); and after 2020, emerging terms such as “management,” “proliferation,” and “attenuates atherosclerosis” indicated a diversification of intervention strategies and analytical approaches (Figure 6).

### 3.4 Reference Burst Analysis and Citation Trajectories

The citation analysis revealed the temporal evolution of research hotspots from 1999 to 2024 through the reference burst detection map. The term “interleukin‐6” (#0) exhibited the strongest burst between 2019 and 2024, indicating its prominence as a current research frontier. “Inflammatory” (#1) and “chlamydia pneumoniae” (#2) showed notable bursts around the early 2000s and approximately 2010, respectively, reflecting peak research attention during those periods. Keywords ranked #3 to #19, including “gut microbiota,” “cd40,” “gene expression profiling,” “mendelian randomization,” “arterial stiffness,” and “vascular events,” represent successive thematic transitions and emerging focal points across different phases.

**FIGURE 7:**
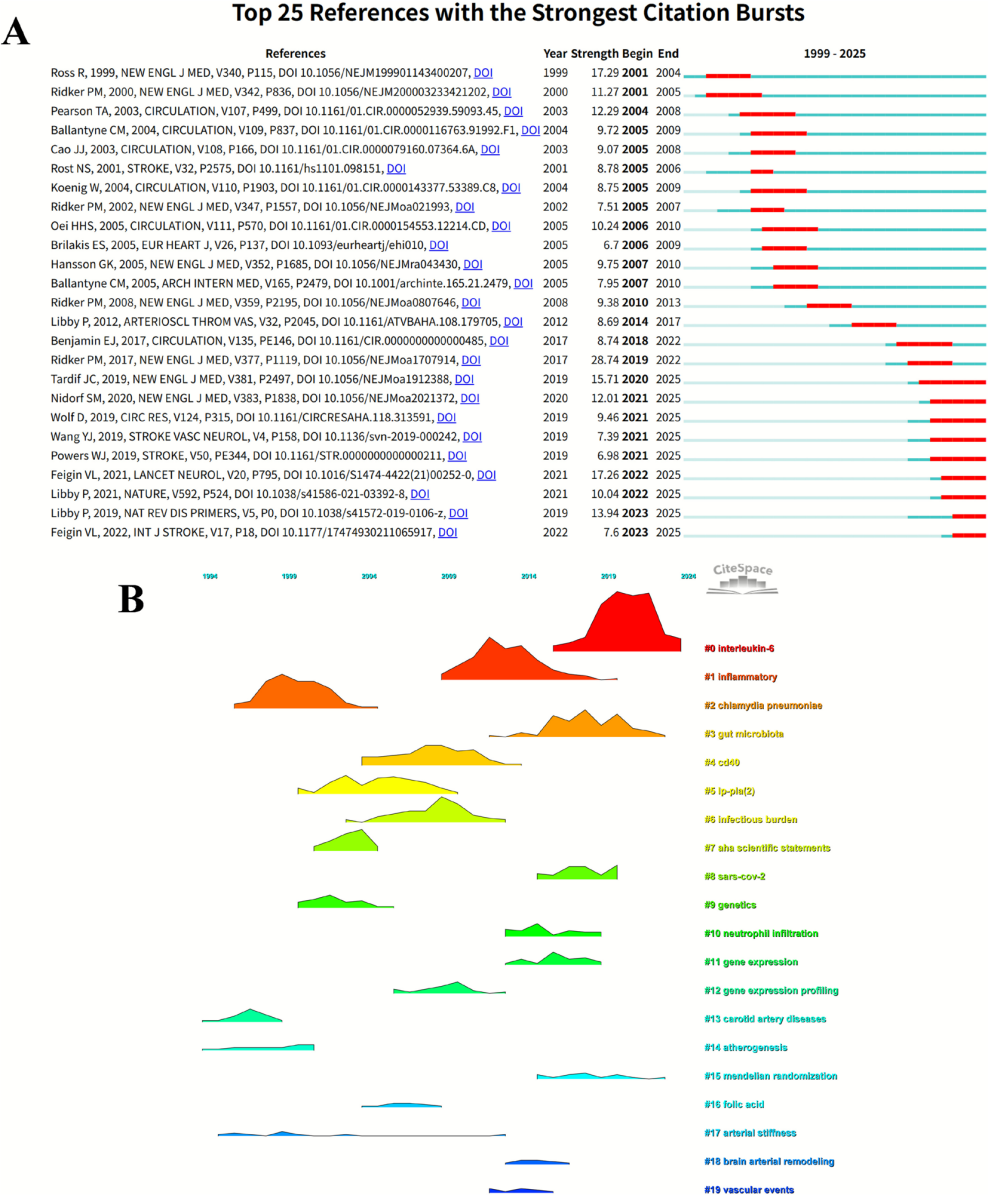
References with strongest citation bursts and thematic evolution. (A) Top 25 references with the strongest citation bursts (1999–2025). (B) Thematic evolution of research clusters over time.

The reference burst strength diagram highlights the top 25 publications with the most significant citation bursts from 1999 to 2025. The work by Ross R (1999) [13] demonstrated the highest burst strength (17.29) during 2001–2004. Multiple influential studies by Ridker PM (2000, 2002, 2008, 2017), Pearson TA (2003), Ballantyne CM (2004, 2005), Cao JJ (2003), Libby P (2012, 2019), and Benjamin EJ (2017) exhibited strong citation bursts in various periods. In the past 5 years, publications by Tardif JC (2019), Nidorf SM (2020), and Feigin VL (2022, 2023) have emerged and sustained citation bursts through 2025, reflecting their ongoing impact in the current research landscape. Collectively, the visualizations illustrate the chronological dynamics and influence patterns of citation hotspots (Figure 7).

### 3.5 Comparative Analysis of Research Output and Thematic Focus: China Versus the United States

#### 3.5.1 Keyword Networks and Thematic Evolution in China and the United States

Visualization based on VOSviewer and CiteSpace demonstrates that both China and the United States have established co‐occurrence networks centered on “inflammation,” “atherosclerosis,” and “ischemic stroke.” In China, keywords such as “activation,” “oxidative stress,” and “macrophages” form modules focused on immune‐inflammatory mechanisms. In the U.S., the network incorporates “c‐reactive protein” and “cardiovascular disease,” extending to clusters including “risk factors,” “coronary heart disease,” and “endothelial dysfunction.” Color‐coded by average publication year, both networks exhibit a temporal shift from macro‐level disease features to molecular mechanisms.

From 2020 to 2025, citation burst analysis highlights differing thematic evolutions. In China, early bursts include “transient ischemic attack,” “cancer,” and “t cells,” followed by “prediction,” “prevention,” and “chain fatty acids,” with later emphasis on “infarction,” “dendritic cells,” and “blood–brain barrier.” In the U.S., initial bursts appear in “cardiovascular risk factors,” “cerebrovascular disease,” and “oxidative stress,” with subsequent focus on “depression,” “macrophages,” “carotid stenosis,” and “cerebral infarction.”

**FIGURE 8:**
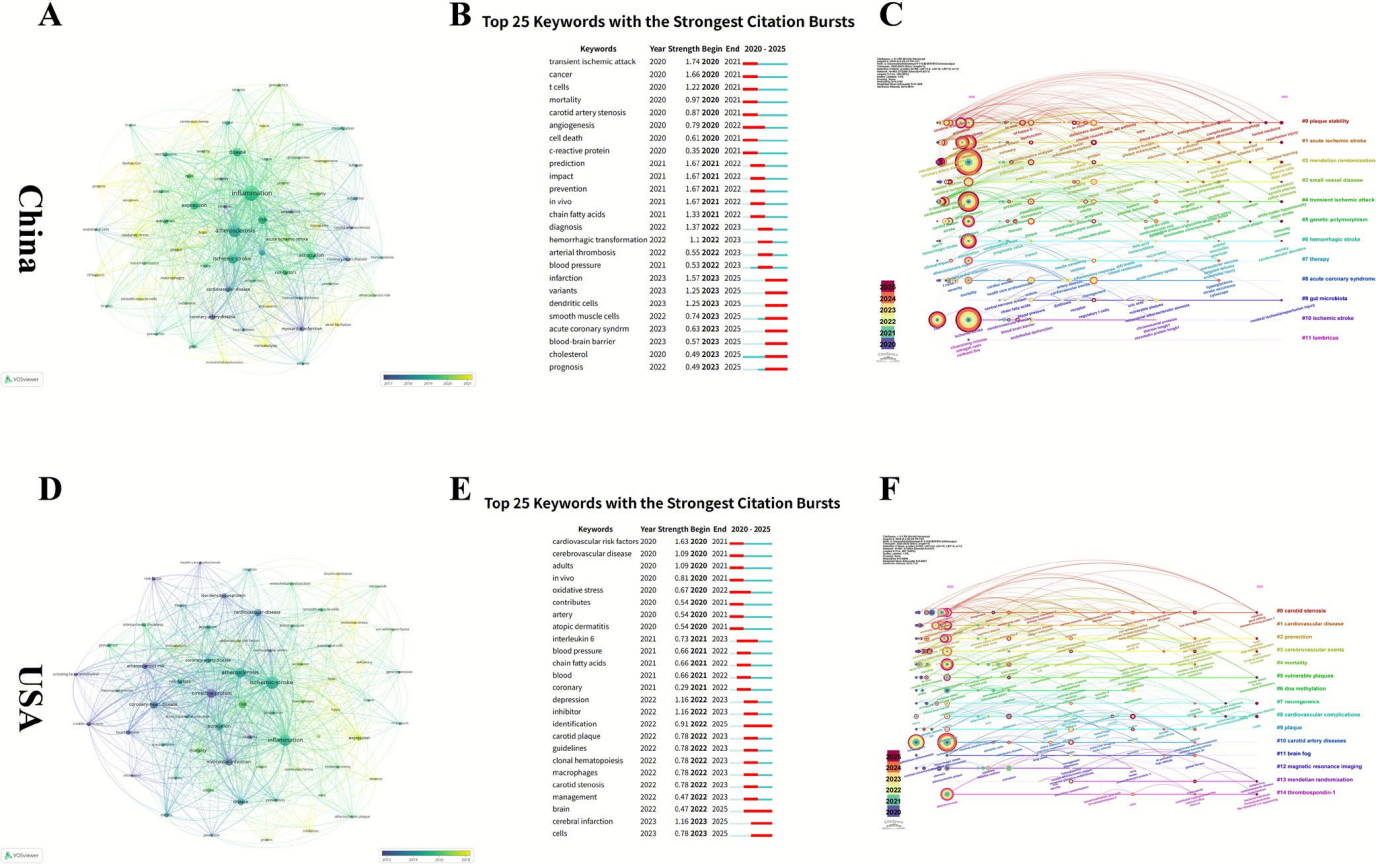
Keyword networks, citation bursts, and temporal evolution in China and USA. (A) Keyword co‐occurrence network in China. (B) Top 25 keywords with the strongest citation bursts in China (2020–2025). (C) Temporal evolution of keyword clusters in China. (D) Keyword co‐occurrence network in the USA. (E) Top 25 keywords with the strongest citation bursts in the USA (2020–2025). (F) Temporal evolution of keyword clusters in the USA.

Timeline clustering reveals 12 major clusters in China, led by Cluster #0 (“plaque stability”), followed by clusters on cerebrovascular disease, genetic epidemiology, gut microbiota, and traditional medicine. The U.S. shows 15 clusters, with Cluster #0 (“carotid stenosis”) most active, and others encompassing cardiovascular prevention, epigenetics, neurogenesis, and imaging. Both countries exhibit a progression from clinical and epidemiological topics to molecular mechanisms and interdisciplinary research, with color gradients indicating a coherent trajectory of thematic evolution (Figure 8).

#### 3.5.2 Multidimensional Keyword Structures and Temporal Shifts: China vs. the United States

Multiple correspondence analysis (MCA), keyword frequency distribution, and temporal trend mapping reveal that both Chinese and U.S. research center on ischemic stroke, atherosclerosis, and inflammation, forming structured multi‐dimensional keyword networks. In the MCA map, Chinese studies span four quadrants—clinical, cellular, epidemiological, and molecular—anchored by “ischemic stroke,” “atherosclerosis,” and “inflammation” (Dim 1: 34.45%, Dim 2: 17.45%). U.S. studies display a denser configuration with keywords clustered around cellular mechanisms, disease progression, prevention, and population traits (Dim 1: 44.36%, Dim 2: 16.45%), with “ischemic stroke” and “cardiovascular risk factors” at the center.

Treemap analysis shows that Chinese research emphasizes “inflammation” (15%), “atherosclerosis” (10%), and “ischemic stroke” (7%), while U.S. research features “ischemic stroke” (11%), “inflammation” (9%), and “atherosclerosis” (7%), along with broader terms like “c‐reactive protein” and “cardiovascular disease.”

**FIGURE 9:**
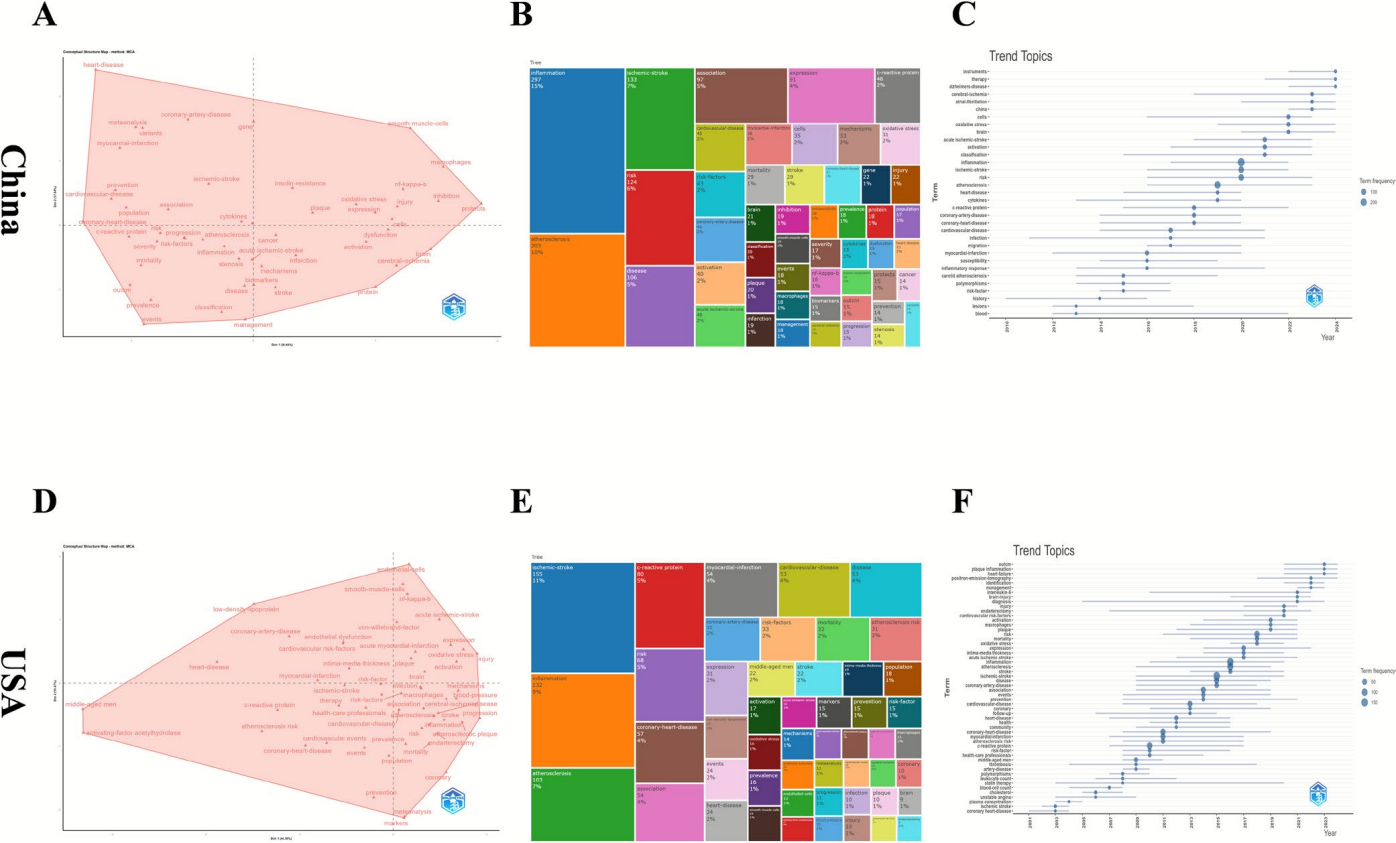
Conceptual structures, thematic distributions, and topic trends in China and USA. (A) Conceptual structure map for China. (B) Keyword tree map for China. (C) Trend topics over time for China. (D) Conceptual structure map for the USA. (E) Keyword tree map for the USA. (F) Trend topics over time for the USA.

Temporal mapping shows that Chinese research intensified after 2010, evolving from risk‐related terms to mechanistic focuses by 2023. U.S. research began in 2001 with cardiovascular risk, shifted to inflammation and demographics by 2010, and after 2015 focused on stroke mechanisms and biomarkers (Figure 9).

### 3.6 Clinical Relevance Assessment Based on Human‐Only Studies From MEDLINE

#### 3.6.1 Keyword co‐Occurrence and Emerging Themes in MEDLINE Human Studies

The VOSviewer keyword co‐occurrence map revealed “stroke,” “atherosclerosis,” “inflammation,” and “ischemic stroke” as central nodes, forming the structural core of the research network. Surrounding terms such as “myocardial infarction,” “cardiovascular disease,” and “cytokines” highlight the integration of vascular and immune pathways, while peripheral keywords including “interleukin‐6,” “Mendelian randomization,” and “oxidative stress” extend the thematic scope toward molecular and genetic mechanisms. Temporal gradients indicate a post‐2018 shift toward emerging research directions, establishing a multidimensional framework centered on stroke and atherosclerosis.

Burst detection from 2020 to 2025 identified 25 high‐intensity keywords. In 2020, emphasis was placed on “microRNAs” and “cryptogenic stroke.” By 2021, terms such as “COVID‐19” (strength 1.45), “innate immunity,” and “carotid artery disease” gained prominence. In 2022, the focus expanded to “insulin resistance,” “platelets,” and “blood–brain barrier,” followed in 2023 by “cytokine” (1.49), “meta‐analysis,” and “lipoprotein(a),” reflecting increased attention to inflammatory regulation and risk stratification.

**FIGURE 10:**
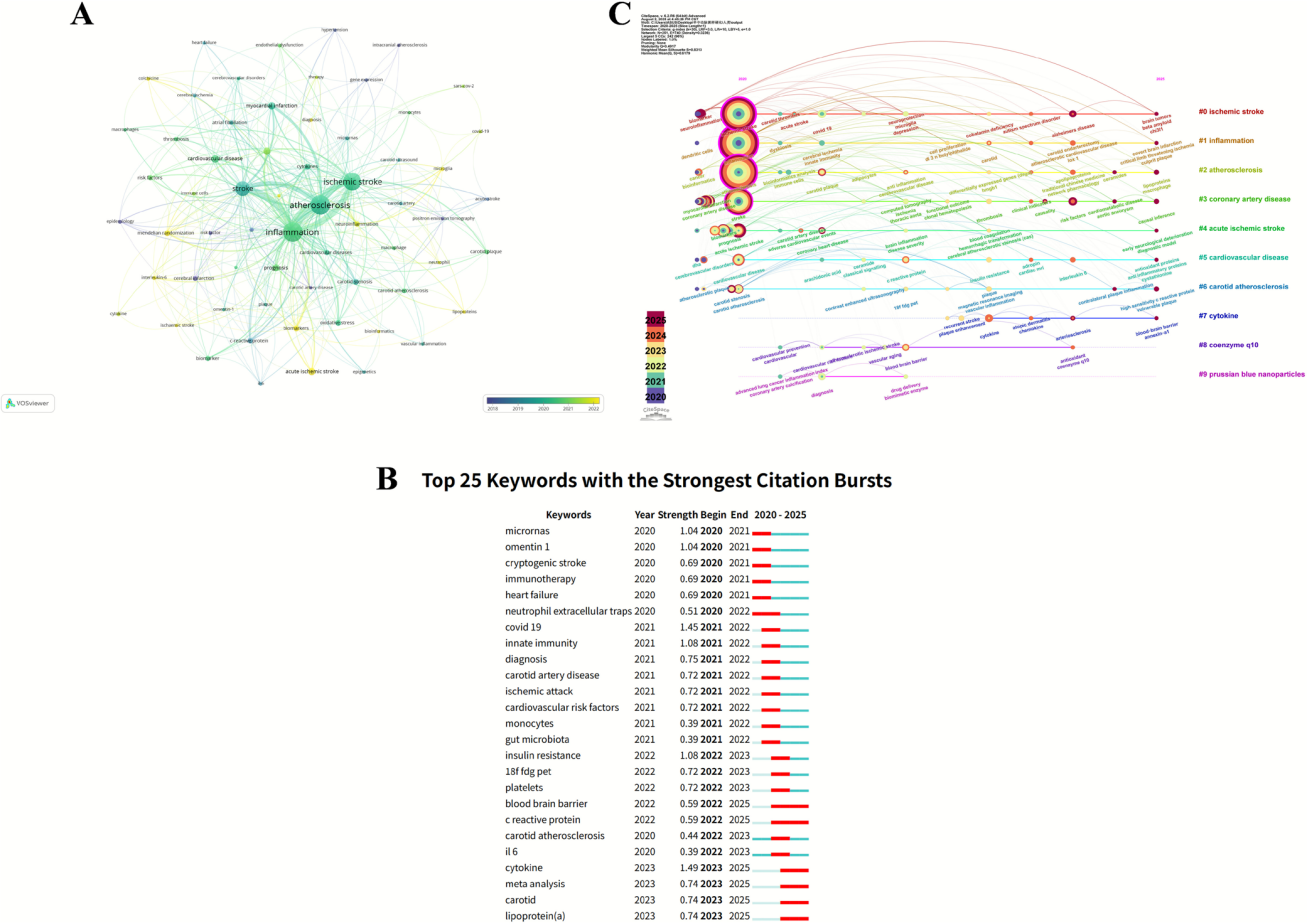
Keyword networks, citation bursts, and temporal evolution in human studies. (A) Keyword co‐occurrence network (human studies). (B) Top 25 keywords with the strongest citation bursts in human studies (2020–2025). (C) Temporal evolution of keyword clusters in human studies.

CiteSpace clustering identified ten thematic groups, including “#0 ischemic stroke,” “#1 inflammation,” and “#2 atherosclerosis.” Research between 2020 and 2022 focused on clinical and mechanistic themes, while post‐2023 trends emphasized precise molecular targeting and interdisciplinary convergence (Figure 10).

#### 3.6.2 Conceptual Structure and Temporal Trends in Human‐Oriented Research

The conceptual structure map based on Multiple Correspondence Analysis (Dim 1: 16.65%, Dim 2: 12.94%) outlines a multidimensional thematic space. Core terms—“inflammation,” “stroke,” “atherosclerosis,” and “ischemic stroke”—are surrounded by “biomarkers,” “cytokine,” “oxidative stress,” and “diagnosis.” The right quadrant centers on neuroinflammatory mechanisms (“microglia,” “gene expression”), the left on diagnostic imaging (“carotid ultrasound,” “magnetic resonance imaging”), the upper on immune regulation (“interleukin‐6,” “Mendelian randomization”), and the lower on exogenous factors (“COVID‐19,” “SARS‐CoV‐2”).

The tree map highlights “atherosclerosis” (132), “inflammation” (130), “ischemic stroke” (107), and “stroke” (77) as dominant terms, with peripheral keywords addressing disease progression (“cardiovascular disease,” “prognosis”) and mechanistic extensions (“Mendelian randomization,” “oxidative stress”). The structure reflects a cerebrovascular‐centered radial network.

**FIGURE 11:**
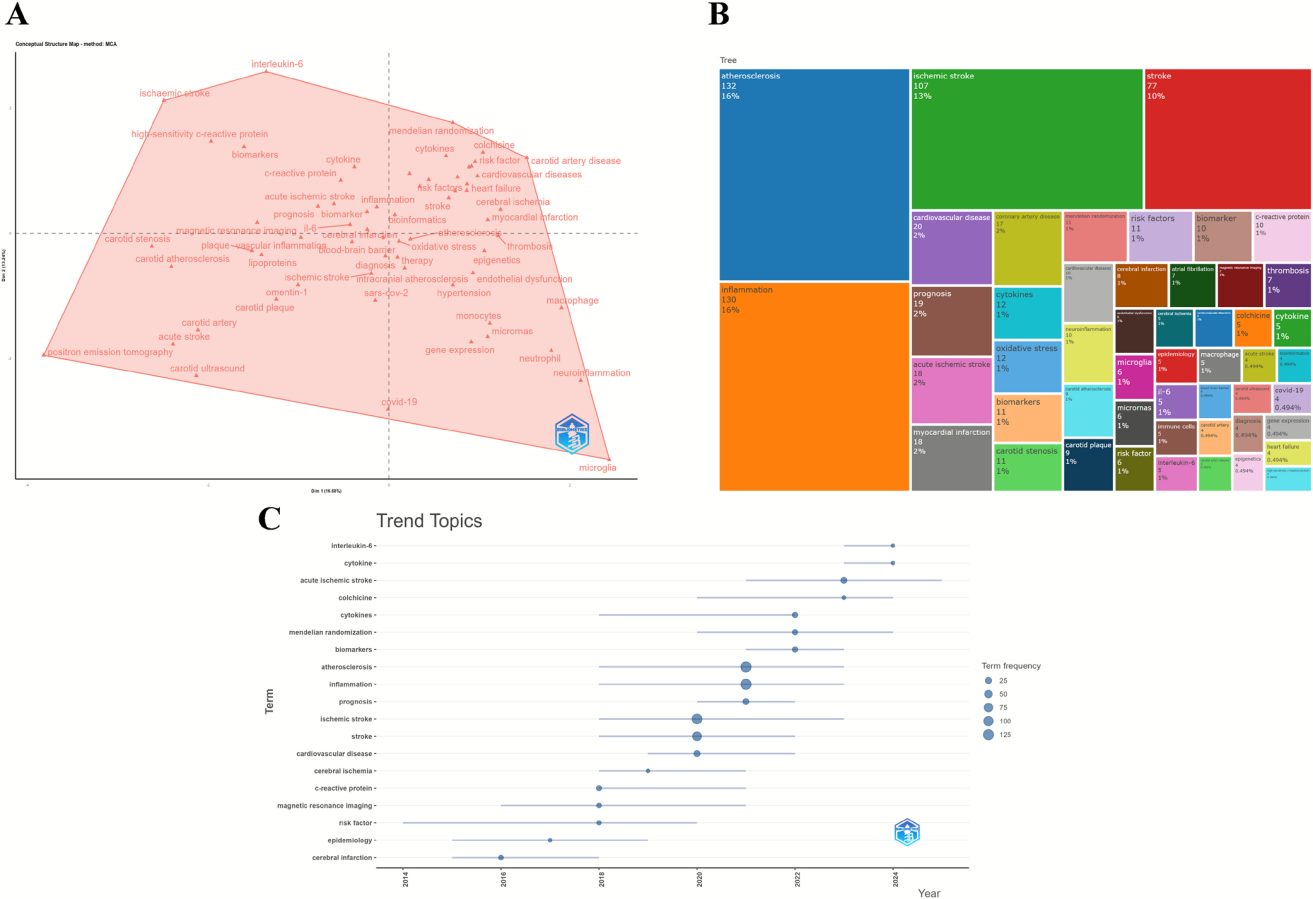
Conceptual structures, thematic distributions, and topic trends in human studies. (A) Conceptual structure map for human studies. (B) Keyword tree map for human studies. (C) Trend topics over time in human studies.

Trend analysis (2014–2024) shows core terms peaking around 2020, followed by increased prominence of “biomarkers,” “Mendelian randomization,” and “cytokines.” Post‐2022, keywords such as “acute ischemic stroke” and “interleukin‐6” indicate a shift toward molecular and inflammatory pathways (Figure 11).

## 4 Discussion

This bibliometric study provides the first comprehensive synthesis of immune‐inflammatory research linking atherosclerosis and ischemic stroke, revealing a global shift from associative to mechanistic and translational investigations.

### 4.1 Overall Analysis of WOS Core Collection

#### 4.1.1 Publication Trends and Collaboration

Research on immune‐mediated links between atherosclerosis and ischemic stroke has grown steadily from 1999 to 2025 (approximately 11% average annual increase), peaking in 2022 before a slight recent stabilization. This body of work spans over 1700 publications in 600+ journals, indicating extensive breadth. The community is highly collaborative, with an average of about 7–8 co‐authors per paper and roughly 22% of publications involving international co‐authorship. Such metrics reflect a multidisciplinary, globally engaged field. The citation impact is strong (around 40 citations per paper on average), and the moderate average publication age (~8 years) suggests that even older studies remain influential. Overall, these statistics portray a maturing field with expanding output, broad participation, and sustained scholarly impact.

#### 4.1.2 Core Research Themes and Network

Keyword co‐occurrence analysis highlights inflammation as the central mechanism connecting atherosclerosis and stroke. In the global network, “inflammation” is the most frequent and strongly linked term [14], closely tied to “atherosclerosis” and “ischemic stroke.” [15] These core concepts form a dense cluster with related keywords such as “C‐reactive protein” (an inflammatory biomarker), “myocardial infarction,” and “cardiovascular disease.” This indicates that researchers approach the problem from multiple angles, linking systemic immune processes to both coronary events and cerebrovascular events. The prominence of inflammatory markers alongside clinical endpoints suggests a deliberate effort to bridge mechanistic insights with clinical observations. The thematic map of the field shows a multi‐dimensional structure: major themes like inflammation and atherosclerosis are well‐developed and central, while broad cardiovascular risk factors provide background context. Smaller, niche topics (e.g., specific genetic polymorphisms) are developed within their subfields but less connected to the main network. A few emerging topics occupy peripheral positions; notably, “neuroinflammation” and “COVID‐19” have recently appeared near the core, indicating that new scientific findings and real‐world events are being incorporated into the central framework. In sum, the field is built on a stable triad of immune–vascular–neurological concepts, yet it remains adaptable and open to novel developments.

#### 4.1.3 Shifting Focus Over Time

The research focus has progressed from broad associations to specific mechanisms and toward clinical translation. Early 2000s: Studies often explored general links and hypotheses—for example, whether infections [16, 17] (e.g., *Chlamydia pneumoniae* , *Helicobacter pylori* ) or classic risk factors (like dyslipidemia) might connect atherosclerosis and stroke [18]. Around 2010: With the acceptance of atherosclerosis as an inflammatory disease, attention shifted to particular pathways such as oxidative stress, endothelial dysfunction, and distinguishing subtypes of ischemic stroke. Mid‐2010s: There was increasing focus on cerebrovascular‐specific mechanisms; for instance, research on atrial fibrillation (a cardiac source of emboli leading to stroke) and activation of various immune cells in stroke became more prominent. 2020 onwards: The trend pivoted further toward translational and analytical approaches. The appearance of terms like “management” suggests a growing emphasis on clinical interventions and strategy, while the rise of “Mendelian randomization” and “causal inference” reflects efforts to move beyond correlations and rigorously test causality between inflammation and stroke. Biomarker discovery also accelerated, with increasing focus on inflammatory mediators (e.g., cytokines, interleukin‐6) as hot topics. Co‐occurrence cluster analysis reinforces this evolution: some clusters center on fundamental vascular injury mechanisms (endothelial cell dysfunction, macrophage‐driven inflammation), others on epidemiological and clinical perspectives (general cardiovascular risk factors, stroke patient cohorts), and a newly emerged cluster is dedicated to genetic and causal analysis (e.g., studies using Mendelian randomization). An unexpected minor cluster even links to neurodegenerative disease (e.g., Parkinson's disease), suggesting a few investigators are examining shared pathways between stroke and other neurological disorders. In summary, the WoS Core Collection analysis depicts a field that has expanded quantitatively and advanced qualitatively—moving from establishing the inflammatory paradigm to unpacking its molecular details and considering how to apply this knowledge in practice.

### 4.2 Comparative Analysis: China vs. United States

#### 4.2.1 Research Output and Impact

China and the United States are the leading contributors to this research area, but with distinct growth patterns and impacts. China's publication output has accelerated sharply in the past decade (especially post‐2015), to the point of rivaling or even surpassing the U.S. in annual papers by the early 2020s. In contrast, the U.S. showed steadier growth from the early 2000s and has maintained a consistently strong presence [19]. The United States still leads in certain impact metrics: its publications have accrued far more total citations, and it serves as the central hub of the global collaboration network. This reflects the U.S.'s longer‐established influence and extensive international partnerships. Thus, China's rise has added considerable new volume and momentum to the field, while the U.S. continues to provide sustained high‐impact contributions and leadership. Both countries are pivotal, with China contributing fresh energy and data, and the U.S. providing foundational impact and global connectivity.

#### 4.2.2 Thematic Focus in Keywords

Both countries share the core trio of inflammation, atherosclerosis, and ischemic stroke as central keywords, yet their networks show different emphases. In China's co‐occurrence network, there is a strong focus on immune cells and oxidative stress mechanisms. Terms such as “macrophages,” “oxidative stress,” “T cells,” and “activation” are prominent, indicating that Chinese research often delves into molecular immunopathology—for example, examining how inflammatory cells and mediators contribute to plaque instability or brain injury during stroke. By contrast, the U.S. keyword network, while also anchored in inflammation and atherosclerosis, extends more into clinical and risk‐factor domains. Keywords like “C‐reactive protein,” “cardiovascular disease,” “risk factors,” and “coronary heart disease” feature strongly in American publications, reflecting an emphasis on epidemiological links and broad cardiovascular risk assessment (e.g., using systemic inflammation markers to predict stroke risk, and situating stroke within the continuum of cardiovascular disease). The U.S. network also includes terms related to outcomes, prevention, and diagnostics—for instance “mortality,” “prevention,” “endothelial dysfunction,” and “magnetic resonance imaging” – suggesting a comprehensive approach from population‐level factors to advanced imaging biomarkers. Notably, temporal analyses show a parallel shift in both countries: earlier studies tended to use broad terms (e.g., “cerebrovascular disease,” “risk factor”), whereas more recent studies on each side highlight specific molecular or cellular terms (such as “inflammasome” and “microRNA” in China, or “oxidative stress” and “macrophages” in the U.S.). This indicates that despite different starting priorities, both research communities have increasingly converged on detailed immune mechanisms in recent years.

#### 4.2.3 Clusters and Evolving Topics

Each country's prominent research clusters underscore its strengths and evolving interests. In China's recent literature, notable clusters include plaque stability [20](emphasizing characteristics of atherosclerotic plaques that trigger stroke, likely through imaging and intervention studies), stroke subtypes and cerebrovascular disease cohorts, genetic epidemiology (leveraging large patient genomic studies emerging from China), gut microbiota and metabolite‐related inflammation, and even a cluster on traditional medicine. The presence of traditional medicine as a research theme reflects a unique aspect of China's contribution—exploring herbal or alternative therapies and their anti‐inflammatory effects on atherosclerosis [21, 22] and stroke [23, 24], an angle less visible in Western research. The United States, in contrast, exhibits a broader array of clusters (around 15 major themes) skewed toward cutting‐edge biomedical and clinical applications. The leading U.S. cluster centers on carotid stenosis, highlighting persistent interest in carotid atherosclerosis as a preventable cause of stroke and efforts to improve its detection and management. Other significant American clusters focus on cardiovascular prevention strategies [25, 26, 27], epigenetic mechanisms in vascular disease (heritable molecular changes affecting atherosclerosis/stroke), neurogenesis and stroke recovery [28] (exploring brain repair processes post‐stroke), and advanced imaging/diagnostic techniques.

Over time, both countries have shifted from predominantly clinical or population‐level topics to more interdisciplinary and mechanistic topics. Chinese research, especially after 2015, moved from general risk factors to detailed immune and biochemical pathways by the early 2020s. The U.S. trajectory similarly evolved: starting from work on traditional cardiovascular risk factors in the 2000s, pivoting around 2010 toward inflammation (and even examining health disparities in stroke risk), then after 2015 homing in on stroke‐specific mechanisms, biomarkers, and targeted therapies.

#### 4.2.4 Complementary Strengths and Collaboration

These patterns illustrate two complementary approaches tackling the same overarching problem. China has brought rapid growth, large‐scale studies, and novel angles (including integrative medicine and microbiome research) in a relatively short time. The United States has built on decades of epidemiological data and clinical trials to drive molecular‐level insights and translational applications. The structural differences in their bibliometric profiles highlight opportunities for cross‐fertilization. For instance, Chinese studies that span clinical data, molecular experiments, and even traditional therapies could benefit from the multi‐dimensional integration and methodological rigor common in U.S. research. Conversely, U.S. research can draw inspiration from some innovative themes emerging in China. Both countries clearly recognize the critical immune‐mediated nexus between atherosclerosis and stroke. Strengthening collaborations between their research communities—combining China's expansive resources and ideas with the U.S.'s deep experience and global networks—would likely accelerate progress in this field.

### 4.3 Analysis Based on MEDLINE (Human‐Only Studies)

#### 4.3.1 Core Concepts in Clinical Context

Focusing on human‐only studies (MEDLINE‐indexed research on patients) confirms that the same core concepts drive clinical investigation. Stroke, ischemic stroke, atherosclerosis, and inflammation remain the dominant keywords, underscoring that immune mechanisms linking atherosclerosis to stroke are central in patient‐oriented research as well. Around this core, the co‐occurrence network features many clinically pertinent topics that intertwine vascular and immune pathways in human disease. For example, “myocardial infarction” and “cardiovascular disease” frequently appear, indicating that stroke is often studied within the broader context of systemic atherosclerotic disease—patients with stroke commonly have coronary disease, and researchers view the immune processes as shared across heart and brain. Similarly, terms like “cytokines,” “interleukin‐6,” and “oxidative stress” surround the core, representing specific inflammatory molecules and pathways examined in patients. The presence of these biomarkers and molecular factors in the network shows that clinical studies actively measure immune mediators (such as circulating cytokine levels or genetic inflammatory markers) alongside clinical observations. In summary, the human‐focused analysis validates the globally identified key themes in a real‐world context: every major link between immunity and vascular disease is being investigated in actual patients—from traditional risk factors to detailed immune biomarkers—to understand and combat stroke [29].

#### 4.3.2 Emerging Clinical Research Trends

Clinical research in recent years has rapidly incorporated new scientific insights and responded to global health events. Keyword burst analysis (2020–2025) highlights several surging topics:

- **2020:** *MicroRNAs* and *cryptogenic stroke* rose in prominence. This reflects an interest in novel biomarkers (microRNAs as circulating indicators or regulators of stroke risk) and efforts to find underlying causes for strokes of unknown origin (cryptogenic strokes).
- **2021:** *COVID‐19*, *innate immunity*, and *carotid artery disease* showed sharp increases. The COVID‐19 pandemic spurred investigations into how systemic infection and inflammation can precipitate strokes or destabilize atherosclerotic plaques. At the same time, there was growing focus on fundamental immune defenses (innate immune responses) in stroke patients, and renewed attention to carotid atherosclerosis management as a key stroke prevention issue.
- **2022:** Terms like *insulin resistance*, *platelets*, and *blood–brain barrier* emerged strongly. This indicates a widening scope: exploring metabolic factors (insulin resistance in diabetes) that exacerbate vascular inflammation, examining platelet activation and thrombo‐inflammation in stroke, and studying how systemic inflammation or vascular pathology compromises the blood–brain barrier during ischemic injury.
- **2023:** *Cytokine*, *meta‐analysis*, and *lipoprotein(a)* became prominent [30]. The emphasis on *cytokines* underscores intense interest in inflammatory mediators as both biomarkers and potential therapeutic targets (for instance, evaluating drugs that block specific cytokines). The appearance of *meta‐analysis* reflects the maturing evidence base—enough clinical studies exist on certain topics (e.g., inflammation markers and stroke risk) to permit comprehensive syntheses and evidence reviews. The rise of *lipoprotein(a)* points to increased examination of this genetically determined lipid risk factor in stroke, likely propelled by new lipid‐lowering therapies targeting Lp(a) and recognition of its role in atherosclerosis beyond the heart [31].

Overall, these trends demonstrate that human studies are highly responsive to new scientific developments and real‐world challenges. The field has been layering greater sophistication onto the core theme of inflammation and stroke—embracing cutting‐edge biomarkers, genetic factors, and analytical methods—all while keeping focus on the ultimate goal of improving stroke prediction, prevention, and treatment.

#### 4.3.3 Multidisciplinary Clinical Approaches

The thematic structure of human studies shows that a wide range of disciplines converge on this medical problem. A multiple correspondence analysis of keywords places the core terms (“inflammation,” “stroke,” “atherosclerosis”) at the center of a multi‐dimensional space, with clusters of related terms around them representing different facets of clinical research:

One cluster is characterized by neuroinflammatory mechanisms (e.g., “microglia,” “gene expression”), suggesting that some clinical studies—perhaps involving advanced imaging or analysis of patient brain tissues and fluids—are examining how immune cells in the brain react during stroke and how gene expression patterns reflect systemic inflammation [32, 33].

Another cluster features diagnostic and assessment tools (terms like “carotid ultrasound,” “magnetic resonance imaging”), indicating significant research aimed at improving the detection of atherosclerotic burden and vascular changes in patients through imaging and other diagnostics [34].

A further cluster connects immune factors with epidemiology and interventions, bringing together terms such as “interleukin‐6” and “Mendelian randomization.” This pairing implies that researchers are using genetic approaches (e.g., variations in IL‐6 signaling as natural experiments) to infer causal links between inflammation and stroke, and are considering targeting such pathways in clinical interventions.

Yet another grouping includes external triggers and systemic conditions (for instance, **“COVID‐19”** and related terms), highlighting that recent studies account for the influence of major infections, environmental factors, and comorbid conditions on stroke via inflammatory pathways.

A keyword frequency treemap further underlines which topics dominate in clinical relevance. The largest blocks are “atherosclerosis” and “inflammation,” each comprising roughly 15%–16% of all keyword occurrences, with “ischemic stroke” and “stroke” together also forming a significant share. This distribution shows that clinical studies give nearly equal weight to the chronic atherosclerotic process and the acute inflammatory response—acknowledging that both aspects are critical to address in reducing stroke risk. Medium‐sized blocks such as “prognosis,” “biomarker,” and “risk factors” reflect practical clinical aims: many studies focus on predicting patient outcomes and identifying measurable indicators of risk or therapeutic response. Smaller blocks (e.g., “oxidative stress,” “microRNA,” “platelets,” and “endothelial dysfunction”) indicate that very specific mechanistic topics do feature in human studies, but usually as subthemes within larger clinical investigations rather than as stand‐alone focal points. Importantly, no single niche topic dominates the landscape; instead, the research appears as a radial network centered on patient‐focused outcomes, with various scientific sub‐disciplines feeding into that center. This means human studies serve as a crucible where insights from basic immunology, cardiovascular medicine, neurology, genetics, and other fields are integrated—all with the goal of improving clinical understanding and care. In sum, looking exclusively at human data accentuates the translational nature of this field: the key discoveries about immune‐mediated atherosclerosis and stroke are being tested and applied in patient‐oriented research, and multiple approaches (from blood biomarkers to imaging to genomics) are being employed to turn this knowledge into better stroke prevention and management [35].

### 4.4 Limitations and Future Directions

#### 4.4.1 Key Limitations

**Fragmented Research Layers:** A notable limitation in the current literature is the fragmentation between basic mechanistic research and clinical studies. Many investigations focus either on molecular immunology (e.g., cytokine signaling, endothelial cell biology, genetics) or on clinical outcomes and risk factors, with relatively few that deeply integrate both aspects. This lack of integration makes it challenging to translate mechanistic discoveries into clinical practice (and vice versa) efficiently.

**Need for Causal Evidence:** Much of the existing research is associative, linking inflammatory markers with stroke risk or observing correlations, without conclusively proving causation. Rigorous causal inference methods (such as longitudinal cohort analyses and Mendelian randomization) and intervention trials have only recently started to gain momentum and remain a smaller portion of the literature. More efforts are needed to discern which immune processes are true drivers of atherosclerosis and stroke (versus just correlated indicators), so that effective targets for therapy can be identified with confidence.

**Underexplored Emerging Topics:** Several promising or novel areas remain underexplored or exist in isolated pockets of research. For example, the influence of the gut microbiota and its metabolites on systemic inflammation and stroke risk is only beginning to be examined and is not yet part of mainstream stroke research. Similarly, neuro‐immune interactions in stroke recovery (how post‐stroke inflammation might affect brain repair or contribute to neurodegeneration) have not been fully integrated into the core literature. A few niche interventions—such as antioxidant supplements or nanoparticle‐based therapies [36] – appear in isolated studies but have not connected with the broader research network. These gaps suggest that potentially important angles (microbiome, neurodegenerative links, novel therapies) have yet to be fully pursued or linked to the central framework.

**Language and Database Bias:** First, restricting the analysis to English‐language publications may have introduced language bias, potentially underestimating research output from non‐English‐speaking regions. Second, using the Web of Science Core Collection (WoSCC) rather than Scopus might slightly affect country‐level rankings and citation metrics. Additionally, the WoSCC dataset did not consistently distinguish between original research and review articles, which may influence keyword and citation patterns. The inclusion of both publication types could slightly overrepresent mature topics while underestimating emerging ones. Future work incorporating multilingual and multi‐database searches could improve the comprehensiveness and reduce potential bias in global comparisons.

#### 4.4.2 Future Research Directions

Foster interdisciplinary collaboration to integrate bench and bedside research. Such collaboration should bring together immunologists, neurologists, cardiologists, and other experts in unified projects. For instance, future studies could embed detailed molecular profiling (immune cell subsets, cytokine measurements, gene expression analyses) within large‐scale clinical stroke cohorts or trials. This bench‐to‐bedside integration will help identify which immunological mechanisms have tangible effects on patient outcomes and can be targeted in interventions.

**Strengthen Causal Inference:** Prioritize research designs that move beyond correlation to establish causation. This includes expanding the use of genetic instruments and Mendelian randomization to test whether specific inflammatory pathways [37] (e.g., IL‐6 signaling, NLRP3 inflammasome activity) are causally linked to stroke risk. Additionally, design and conduct clinical trials for promising anti‐inflammatory or immunomodulatory strategies—for example, testing whether therapies like IL‐6 inhibitors or colchicine (an anti‐inflammatory drug) can reduce stroke incidence or improve outcomes, building on encouraging results from cardiovascular disease trials.

**Broaden Immunological Scope:** Expand investigations to cover a wider range of immune‐related factors beyond the relatively few extensively studied markers (such as C‐reactive protein [38] and IL‐6). Future research should delve into adaptive immunity (the role of T‐cells, B‐cells, and autoimmunity in atherosclerosis), chronic infections or latent pathogens that might fuel vascular inflammation, and other cytokines or immune mediators (e.g., TNF‐alpha, IL‐1, interferons) that could be significant in plaque development or stroke injury. A broader immunological view could uncover new targets and improve understanding of patient subsets who have unusual inflammatory profiles.

**Leverage Advanced Technologies:** Make full use of emerging technologies to gain deeper insights. This includes multi‐omics approaches (integrating genomics, transcriptomics, proteomics, metabolomics) to map out comprehensive inflammatory profiles in patients, advanced imaging techniques (such as vascular PET scans for plaque inflammation or high‐resolution MRI for subtle brain changes) to visualize the disease process, and artificial intelligence/machine learning to analyze big data. Applying these tools can reveal complex risk patterns or biomarker combinations that single‐discipline studies might miss, and could lead to personalized risk prediction models or novel diagnostic criteria based on immune signatures.

**Enhance Global Collaboration:** Utilize the complementary strengths of different countries' research. International collaborations could marry the large patient populations, big data, and unique approaches seen in China (including studies on lifestyle factors or traditional medicines) with the detailed mechanistic experiments and long‐term datasets often available in the US and Europe. Such partnerships would generate more robust, generalizable findings and accelerate innovation. Sharing data and resources globally will also help validate discoveries across diverse populations and healthcare settings [39].

**Explore New Intersections:** Pay attention to and investigate emerging intersections with related fields. For example, examine the overlap between post‐stroke inflammation and neurodegenerative diseases (are stroke survivors with high inflammatory responses at greater risk of dementia or Parkinson's disease, and could modulating inflammation help?). Similarly, study the impact of acute systemic events like pandemics on chronic cardiovascular conditions—COVID‐19 brought this to light, but future research should clarify how severe infections or other inflammatory stressors might trigger vascular events or worsen atherosclerotic disease in the long term. As the population ages and faces new health challenges [40], understanding these connections will be increasingly important.

In summary, by addressing the above limitations and pursuing these strategies, the field can further accelerate its progress. The ultimate goal is to effectively translate the growing understanding of immune‐mediated mechanisms into tangible improvements in stroke prevention and therapy, thereby reducing the burden of atherosclerotic ischemic strokes worldwide [41, 42].

## 5 Conclusion

This bibliometric study offers a comprehensive, multi‐dimensional assessment of global research on immune‐mediated mechanisms linking atherosclerosis and ischemic stroke. By integrating global publication trends, cross‐national comparisons between China and the United States, and clinically focused human studies, the analysis delineates a clear progression toward mechanistic depth, genetic inference, and translational biomarker discovery. China has demonstrated rapid expansion with growing emphasis on cellular and molecular immunology, whereas the United States continues to lead in scholarly impact and clinically oriented research. Human‐specific data further substantiate these thematic evolutions, reinforcing the clinical relevance of immune processes in stroke pathophysiology. Nevertheless, the field remains compartmentalized, with limited integration between basic science and clinical application, underutilization of causal inference methodologies, and insufficient exploration of emerging domains such as the gut–brain axis and neuroimmune regulation. Advancing this field will require enhanced cross‐disciplinary integration, broader immunological scope, and the application of multi‐omics and precision technologies. Deepening international collaboration—particularly between China and the United States—may accelerate methodological innovation and knowledge translation. Ultimately, bridging immune biology and clinical practice holds substantial promise for identifying actionable targets, reducing disease burden [43], and guiding precision strategies for cerebrovascular disease prevention.

## Author Contributions

Hongdong Hao (HH) conceived the study design, performed data analysis, and drafted the initial manuscript. Dian Chen (DC) contributed to data collection, preprocessing, and visualization. Cheng Qian (CQ) assisted with bibliometric analysis and interpretation of results. Xuanyi Zhou (XZ) and Xi Peng (XP) participated in literature review and data validation. Guanlin Wang (GW) supported statistical analysis and figure preparation. Jingjing Tang (JT) contributed to manuscript revision and critical editing. Hai‐Xin Liu (HL) supervised the entire project, provided methodological guidance, and critically revised the manuscript for important intellectual content. All authors read and approved the final manuscript.

## Funding

This work was supported by the National Natural Science Foundation of China Cultivation Project (Grant No. 2024PY‐NS‐027), the Research Project of Traditional Chinese Medicine by the National Administration of Traditional Chinese Medicine (Grant No. 2024ZYYC113), and the Shanxi Provincial Science and Technology Innovation Talent Team (Grant No. 202304051001044).

## Conflicts of Interest

The authors declare no conflicts of interest.

## Data Availability

Data sharing not applicable to this article as no datasets were generated or analyzed during the current study.
